# Effect of* Abelmoschus esculentus* Powder on Ovarian Histology, Expression of Apoptotic Genes and Oxidative Stress in Diabetic Rats Fed with High Fat Diet

**Published:** 2019

**Authors:** Naeem Erfani Majd, Hajar Azizian, Mohammad Reza Tabandeh, Ali Shahriari

**Affiliations:** a *Department of Basic Sciences, Faculty of Veterinary Medicine, Shahid Chamran University of Ahvaz, Ahvaz, Iran. *; b *Department of Biochemistry and molecular biology, Faculty of Veterinary Medicine, Shahid Chamran University of Ahvaz, Ahvaz, Iran. *

**Keywords:** Diabetes, High Fat Diet, Abelmoschus esculentus, Apoptosis, Oxidative stress, Ovary, Histology

## Abstract

Okra (*A.esculentus*) is an antidiabetic plant whose beneficial effect on ovarian dysfunction in diabetes condition has not been clarified. The present study was designed to examine the effect of Okra powder on serum oxidant/antioxidant status, ovarian structure, and expression of apoptotic/antiapoptotic related genes in ovary of experimentally induced high fat diet diabetic rats. Diabetes was induced by 5 weeks feeding of Wistar rats with high fat diet (HFD) and subsequent i.p injection of STZ (35 mg/kg). Diabetic animals (serum glucose above 250 mg/dL) were treated with Okra powder (200mg/kg) supplemented in diet or metformin (200mg/kg) for 30days. After 30 days of treatment, animals were euthanized and insulin resistance markers (insulin and glucose levels and HOMA-IR), ovarian expression of apoptotic/antiapoptotic genes (Bax, caspase3 and Bcl2) and serum oxidant/antioxidant levels (SOD, GPX and CAT activities and MDA level) were determined. The ovaries were also processed for histological study. Hyperglycemia and reduced body weight of diabetic rats were improved after administration of Okra for 30days. This effect was relatively similar to metformin. Okra resulted in reduction of follicular atresia in concomitant with down regulation of apoptotic related genes (Bax and caspase3*) *in ovary of diabetic rats. Okra could also diminished oxidative stress in diabetic rats by increasing of serum GPX and CAT activities and reducing the lipid peroxidation level. The results of the present study revealed that Okra powder could be useful intervention for improvement of ovaian dysfunction in diabetic rat by three probable mechanisms; attenuation of glucotoxicity, down regulation of ovarian apoptosis related genes and reduction of oxidative stress.

## Introduction

Obesity is an abnormal condition accumulating lipid in adipose tissue (1). Based on statistics published by the World Health Organization in 2013, the incidence of obesity has doubled worldwide since 1980 (2). Obesity is caused by various environmental and genetic factors. One of the main environmental factors causing obesity is feeding with the high-fat (HF) diet (3). Obesity is associated with various diseases such as diabetes, cardiovascular diseases and reproduction complications such as subfertility or infertility (4, 5, 1, 6). Obesity is a major contributor to the development of insulin resistance and impaired glucose tolerance (7, 8). Because insulin signalling has a central role in ovarian functions, insulin resistance associated with high calorie diet ingestion can negatively alter the ovarian steroidogenesis, follicular development, and granulosa cell proliferation in female (6, 9, 10, 11). The previous study reported that the risk of infertility in women increases with increasing body mass index value (12). Obese women have been shown to be three times more likely to suffer from infertility compared with women who have normal BMIs (12). Suboptimal oocyte quality has also been noted in obese females. Higher numbers of atretic follicles and lower numbers of antral follicles has been reported in obese female rabbit fed by a HFD containing 83% fat (13).

The major factor underlying the adverse effect of obesity on ovarian function is believed to be oxidative stress. In female, oxidative stress had adverse effect on ovarian follicular survival, folliculogenesis, and oocyte health (14). Depletion of ovarian antioxidants enhances the initiation of apoptosis in ovary by regulation of apoptotic signaling modulators including the pro-apoptotic (Bax, Bcl-XShort) and anti-apoptotic (Bcl-2, Bcl-XLong, Mcl-1) proteins (15, 16). 

Because current evidence suggests that oxidative stress can have significant negative impacts on female fertility, nutritional or pharmacological intervention may prove to be effective strategies to protect female fertility.

Natural antioxidants, which are ubiquitous in many medicinal plants, have also received great attention and have been used extensively to improve metabolic complications in diabetic patients (17, 18, 19, 20). Although many of traditionally employed antidiabetic medicinal plants may improve female potential and ovarian functions (21), the underlying mechanisms are not fully determined. Metformin is the most commonly used drug in T2DM. Metformin works by improving the sensitivity of peripheral tissues to insulin (22, 23), which results in a reduction of circulating insulin levels. Metformin inhibits hepatic gluconeogenesis and it also increases the glucose uptake by peripheral tissues and reduces fatty acid oxidation (24). Okra (*Abelmoschus esculentus*), synonym of *Hibiscus esculentus* also called as lady’s finger is a tropical vegetable belonging to the mallow family (25). *A.esculentus*is rich in carbohydrates, minerals and vitamins is also a source of flavonoid compounds that have antioxidant activity (26). Recent study reported that the extract of okra lowers blood glucose and serum lipids in high-fat diet-induced obese mice (27, 28). Ethanol extract of okra and its major flavonoids isoquercitrin and quercetin 3-O-gentiobioside reduces blood glucose and serum insulin levels and improves glucose tolerance in obese mice (28). The antioxidant activity of seed extracts of *A.esculentus *has been reported in the literature (29, 30, 31). It has been reported that the peel and seed powders of *A.esculentus *play antidiabetic and antihyperlipidemic roles in streptozotocin-induced diabetic rats (32).

In spite of beneficial actions of* A.esculentus *for treating the metabolic complications in diabetic patients, its impact on ovarian health in diabetes condition has not been clarified. The present study was designed to examine the effect of *A.esculentus* powder on ovarian histological changes, oxidant/antioxidant status, and expression of apoptotic/anti apoptotic related genes in ovary of experimentally induced insulin resistance rats.

## Experimental

*Preparation of A.esculentus powder*

*A.esculentus* was collected in the October 2014 from the local farm in Ahvaz, South-West of Iran. The plants were kindly identified by Plant Taxonomy laboratory, Faculty of Agriculture Science, Shahid Chamran University of Ahvaz. The fruits (seed and peel) were washed with water and shade dried at room temperature. The dried materials were grounded into fine powder using a mixer grinder and then was weighed and kept well from moisture in a plastic vials in desiccator for further use.

*Measurement of flavonoids content (FC)*

The FC was determined using the method suggested by Huang *et al.* (33) with minor modifications. Five mL of 2% aluminium trichloride (AlCl3) in methanol was mixed with the same volume of the* A.esculentus* powder (0.4 mg/ mL). Absorption of the resulting solution was read at 367 nm using UV-visible spectrophotometer (BioTek, CA, USA) against a blank sample containing 5 mL powder solution with 5 mL methanol without AlCl3. The FC was determined using a standard curve with quercetin (0.2-1 mg/mL) as the standard. FC was expressed as mg of quercetin equivalents per 100 gr powder. All experiments were performed in triplicate.

*Measurement of phenolic content (PC)*

The samples were measured for PC colorimetrically using the Folin-Ciocalteu method (34) with slight modifications. A 100 μL of *A. esculentus *was mixed with 0.5 mL Folin-Ciocalteu reagent (diluted 10 times with distilled water). 7 mL of distilled water was added to the solution and it was allowed to stand at room temperature for 5 min. Then, 1.5 mL sodium bicarbonatesolution (60 mg/ mL) was added to the mixture and left at room temperature in dark place for 2 h. Absorbance was read at 725 nm against blank using UV-visible spectrophotometer (BioTek, CA, USA). A calibration curve was constructed using a standard solution of gallic acid (0.2-1 mg/mL). Results were expressed as mg gallic acid per 100 gr powder. All experiments were performed in triplicate.

*Animals and diets*

Healthy adult Female Wistar rats (200–220 g) were obtained from the experimental animal holding of Joundishapour University of Medical Sciences, Ahvaz, Iran. The animals were housed in standard cages and maintained under controlled room temperature (23 ± 1 °C) and humidity with 12/12 h light/dark cycle. They were provided rat chow (Pars, Tehran, Iran) and water at libitum. All animals used were cared for according to the guide for the care and use of laboratory animals by the national academy of sciences (National Institutes of Health publication No. 86-23). Initially, all rats were housed in conventional conditions and fed standard diet and water ad libitum at the animal facility (Research Resource Facility) for 1 week before experiments began. 

Animals were divided into 5 equal groups and each group consisted of five rats as follow:

Group I: Rats were fed by standard diet.

Group II: HFD diabetic rats

Group III: HFD diabetic rats received* A. esculentus* (200mg/kg)

Group IV: HFD diabetic rats received metformin (200 mg/kg). 

Group V: Rats received normal diet and *A.esculentus* (200mg/kg)

Groups II, III and IV were fed by high fat (HF) diet for 5 weeks, whereas Groups I and V consumed normal diet for the same period. The fat content of HFD was adjusted to 60% by addition of beef tallow into normal diets (35), Normal diet contained pellet chow of standard composition containing all the recommended macro and (56% carbohydrate, 18.5% protein, 8% fat, 12% fiber and adequate levels of minerals and vitamins). After 5-weeks feeding of the HF diets, rats were treated with a single dose of STZ (35 mg/kg, i.p) that was prepared in citrate buffer 0.1 M, pH = 4 (Sigma, Germany). Five days after STZ treatment glucose was measured by hand-held glucometer (Medisign, China) and diabetes induction was confirmed if serum glucose of rats was above 150 mg/dL. The next day after diabetes confirmation was considered as day 0 of treatment. After confirmation of diabetes incidence, animals of groups III and V were treated orally with *A.esculentus* powder at dose of 200 mg/kg body weight (about 30mg/kg total phenol plus 30 mg/kg total flavonoids) for 30 days (32) and group IV was orally treated with metformin (200 mg/kg) for the same period. Animals in groups II, III, and IV were maintained on a high calorie diet throughout the experiment, while rats in groups I and V had access to standard diet during the experiment. The body weight was determined every week. 

*Serum biochemical assays*

After overnight (12 h) fasting, the rats were anesthetized using ketamine and xylazine (100 mg/kg of ketamine and 10 mg/kg of xylazine) at day 30 after treatment. Heart blood samples were collected, and sera were separated and stored at -20 °C for use. Serum glucose was measured using commercial kit (Pishtazteb, Iran) as recommended by the manufacturer. Insulin concentration was measured using a species ELISA kit (KOMA BIOTECH INC, Sout Korea) in a multiplate ELISA reader (BioTek, CA, USA) based on recommended protocol by the manufacturer.

*HOMA-IR estimation*

The homeostasis model assessment of basal insulin resistance (HOMA-IR) was used to calculate an index from the product of the fasting concentrations of plasma glucose (mmol/L) and plasma insulin (μU/mL) divided by 22.5. Lower HOMA-IR values indicated greater insulin sensitivity, whereas higher HOMA-IR values indicated lower insulin sensitivity (insulin resistance) (36).

**Table 1:** Characteristics of primers which were used for Real-Time PCR analysis

**GenBank**	**Size (bp)**	**Sequences**	**Gene name**
NM_017059.2	145	F: TGCTACAGGGTTTCATCCAG R: TGTTGTTGTCCAGTTCATCG	Bax
NM_016993.1	135	F:ATCGCTCTGTGGATGACTGAGTAC R:AGAGACAGCCAGGAGAAATCAAAC	Bcl2
XM_006253130.3	181	F: AATTCAAGGGACGGGTCATG R: CAGATCCCGTGTATTGTGTCA	Caspase3
XM_017593963.1	119	F: AGTTCAACGGCACAGTCAAG R: TACTCAGCACCAGCATCACC	GAPDH

F: forward sequence R: reverse sequence.

**Table 2:** Serum insulin level and HOMA-IR in diabetic rats after treatment by *A. esculentus *powder and metformin. Values are mean ± SD, n = 5 animals per group. Different letters in each column denote significant differences (*P *< 0.05).

**Group**	**Insulin (pg/mL)**	**HOMA-IR (mmol/L** * **μIU/L)**
Normal Diet Control	87.08 ± 9.2 a	10.56 ± 3.32 a
HFD*	47.9 ± 3.2 b	21.77 ± 1.45 b
HFD + A. esculentus	62.06 ± 3.96 c	17.8 ± 4.47 c
HFD + Metformin	51.05 ± 6.09 b	15.86 ± 4.82c
Normal Diet + A. esculentus	65.83 ± 9.48 c	8.62 ± 0.54 d

* HFD: High Fat Diet + STZ treatment.

**Table 3:** Serum MDA level and SOD, GPX and CAT activities in diabetic rats after treatment by *A. esculentus *powder and metformin. Values are mean ± SD, n = 5 animals per group. Different letters in each column demonstrate significant difference (*P *< 0.05)

**Group**	**MDA (μmol/L)**	**SOD (IU/mL)**	**GPX (IU/mL)**	**Catalase (IU/mL)**
Normal Diet Control	0.869 ± 0.28 a	1.552 ± 0.06 a	4.628 ± 0.73 a	2.899 ± 0.18 a
HFD*	1.280 ± 0.08 b	1.62 ± 0.01 a	5.1828 ± 1.10 a	1.466 ± 0.47 b
HFD + A. esculentus	0.837 ± 0.06 a	1.542 ± 0.03 a	7.848 ± 0.91 b	4.258 ± 0.81 c
HFD + Metformin	1.045 ± 0.09 a	1.667 ± 0.06 a	3.987 ± 1.06 a	2.473 ± 0.47 a
Normal Diet + A. esculentus	1.44 ± 0.36 c	1.556 ± 0.03 a	4.537 ± 1.44 a	1.444 ± 0.29 b

* HFD: High Fat Diet + STZ treatment.

**Table 4:** Mean number of atretic follicles present in the ovaries of diabetic rat after treatment with *A. esculentus *powder. Different letters denote significant differences (*P *< 0.05).

**Group**	**atretic preantral follicle**	**atretic antral follicle**
Normal Diet Control	0.6725 ± 0.09 a	0.7 ± 0.3 a
HFD*	2.16 ± 0.29 b	2.96 ± 0.3 b
HFD + A. esculentus	1.203 ± 0.07 c	1.43 ± 0.5 c
HFD + Metformin	1.376 ± 0.13 c	2.28 ± 0.29 b
Normal Diet + A. esculentus	1.18 ± 0.09 c	0.825 ± 0.58 a

HFD: High Fat Diet + STZ treatment.

**Figure 1:**
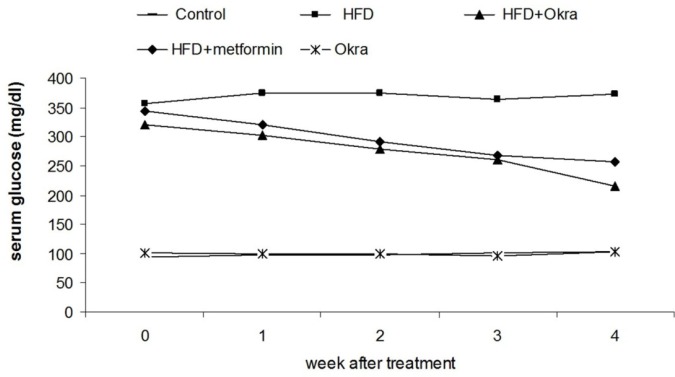
Serum glucose level in diabetic rats after treatment with *A. esculentus *powder and metformin. Values are mean ± SD, n = 5 animals per group

**Figure 2:**
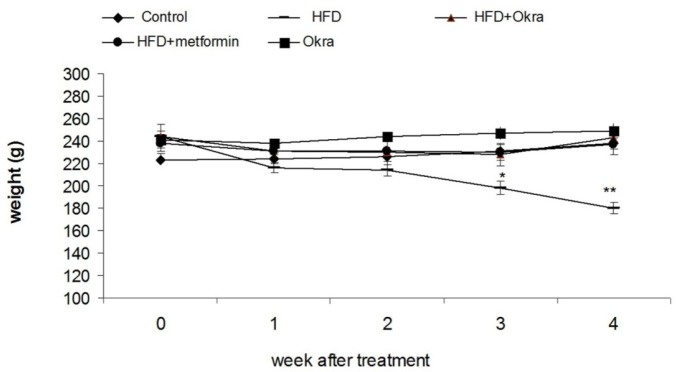
Body weight changes of diabetic rats after administration of *A. esculentus *powder and metformin. Values are mean ± SD, n =5 animals per group. Different numbers of * showed significant difference (*P *< 0.05) are

**Figure 3:**
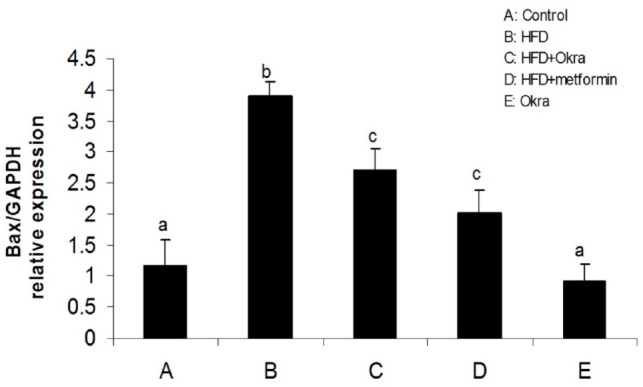
mRNA level of Bax gene in ovary of diabetic rats after treatment with *A. esculentus *powder and metformin. Data were presented as the mean ± SD. Different letters denote significant differences (*P *< 0.05).

**Figure 4:**
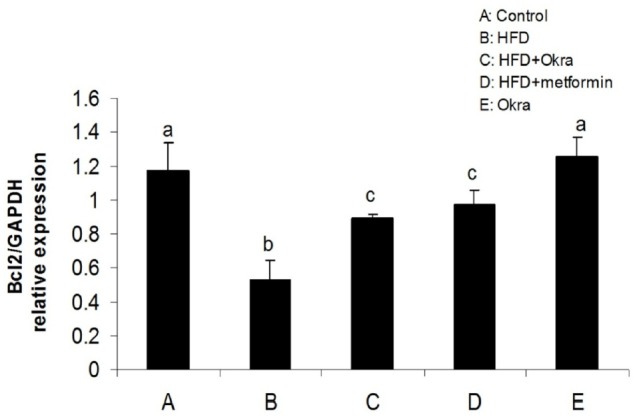
mRNA level of Bcl2 gene in ovary of diabetic rats after treatment with *A. esculentus *powder and metformin. Data were presented as the mean ± SD. Different letters denote significant differences (*P *< 0.05).

**Figure 5:**
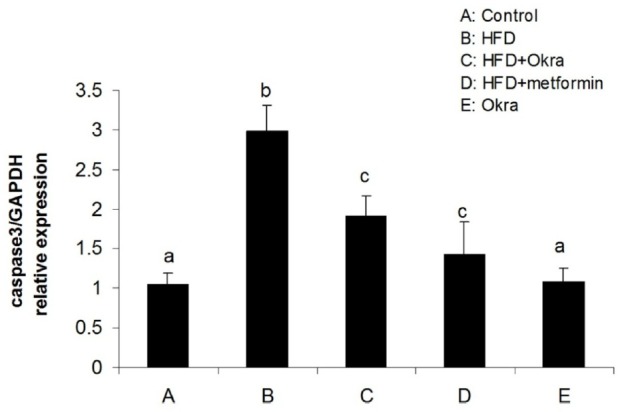
mRNA level of Caspase3 gene in ovary of diabetic rats after treatment with *A. esculentus *powder and metformin. Data were presented as the mean ± SD. Different letters denote significant differences (*P *< 0.05)

**Figure 6:**
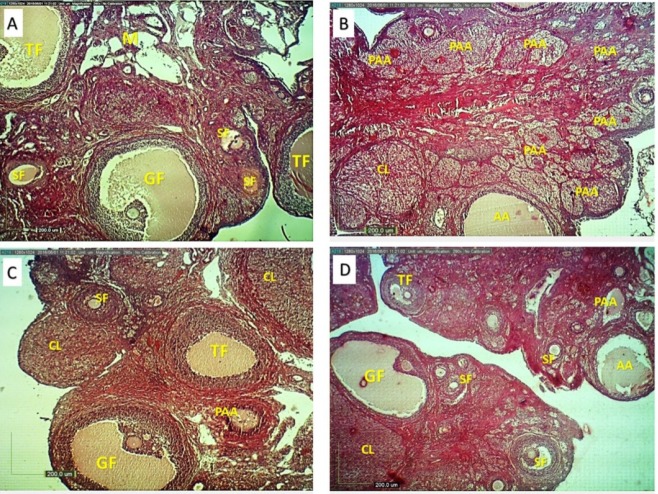
Ovarian structure in different groups (H&E, ×4). Normal rats showed developing ovarian follicles in different stages (A), HFD diabetic rats showed more preantral and antral atretic follicles (B). *A. esculentus *and Metformin treated rats (C and D) showed decreased number of preantral and antral atretic follicles and increased the number of antral follicles and growing CL. SF: secondary follicle, TF: tertiary follicle, G:graafian follicle, PAA: preantral atretic and AA: antral atretic follicles, CL: corpus luteum, M: medulla

*Determination of serum antioxidant enzymes activities*

The activity of superoxide dismutase (SOD) (RANSOD, Randox Com, UK) and Glutathione peroxidase (Gpx) (RANSEL, Randox Com, UK) were estimated using the commercial available kits. SOD and Gpx activities were expressed by the unit of U/Lof serum.

Serum catalase activity was measured according to the method of Aebi (37). The reaction was initiated by the addition of hydrogen peroxide to the reaction mixture and the level of enzyme activity was quantitated according to the ability of the serum catalase to decompensate hydrogen peroxide by monitoring the decrease in absorbance at 240 nm against a blank contains phosphate buffer instead of substrate. 

The value of log A1/A2 for a measured interval was used for unit definition owing to the first-order reaction of enzyme. One unit of CAT is the amount of enzyme that decomposes 1.0 μmole of hydrogen peroxide per min at pH 7.0 and 25 °C.

*Measurement of lipid peroxidation*

The level of lipid peroxidation was indicated by the content of malondialdehyde (MDA) in serum. Serum MDA was determined using the thiobarbituric acid reactive substance (TBARS) assay, as described by Buege and Aust (38) with slight modifications. Briefly, 100 uL of serum was mixed thoroughly with 200 uL of a stock solution of 15% w/v trichloroacetic acid, 0.375% w/v thiobarbituric acid and 0.25 M hydrochloric acid. The solution was sanded for 15 min in a boiling water bath. After cooling, the precipitate was removed by centrifugation at 5000 rpm for 10 min. The absorbance of the clear supernatant was determined at 535 nm and MDA concentration calculated using 1.56 × 10^5^ mol^ -1^ cm^-1^ as molar absorbance coefficient. MDA results were expressed as nmol/mL of serum.

*RNA isolation and cDNA synthesis*

At the end of the experiment, animals were scarified and left ovary was immediately collected and frozen at -70 °C. Total RNA was isolated using RNX ^TM ^reagent according to the manufacturer’s procedure (CinnaGen, Iran). Concentration of extracted RNA was calculated at a wavelength of 260 nm using nano drop spectrophotometry (Eppendorf, Germany). To detect the purity of RNA, its optical density (OD) absorption ratio at 260/280 nm was determined and samples having a ratio more than 1.8 were used for cDNA synthesis. Reverse transcription was carried out with the Rocket Script RT PreMix kit using 1 µg of RNA and oligo dT based on manufacturer’s protocol (Bioneer Corporation, South Korea). Reverse transcription was carried out at 42 °C for 90 min followed by incubation at 80 °C for 3 min. cDNAs were stored at -20 °C until used in the real-time PCR.

*Real-time quantitative RT-PCR*

To evaluate the expression levels of Bax, Bcl2 and caspase3 in ovary, real-time PCR analysis was performed using qPCR^TM^ Green Master Kit for SYBR Green I^®^ (Jena Biosciense, Germany) on a Lightcycler^®^ Detection System (Roche, USA). Relative expression level of the Bax, Bcl2 and caspase3 transcripts were compared to rat GAPDH as housekeeping gene. Specific sets of primers (Bioneer, South Korea) designed for this study is shown in table 1. Reactions were performed in a 12.5 μL mixture containing 6.25 μL qPCR^TM^ Green Master Kit for SYBR Green I^®^ (Jena Biosciense, Germany), 0.25 μL of each primer (200 nM), 3 μL cDNA (100 ng) and 2.25 μL nuclease-free water. The PCR protocol used consisted of a 5 min denaturation at 94 °C followed by 45 cycles of 94 ^o^C for 15 sec, 60 ^o^C for 30 sec. Reactions were performed in triplicate. Two separate reactions without cDNA or with RNA were performed in parallel as controls. Relative quantification was performed according to the comparative 2^-ΔΔCt^ method and using Lightcycler 96^®^ software. Validation of assay to check that the primer for target genes and GAPDH had similar amplification efficiencies was performed as described previously. All qPCR analysis was performed according to the Minimum Information for Publication of Quantitative Real-Time PCR Experiments (MIQE) guideline (39).

*Morphological study of ovaries*

The ovaries were dissected and fixed in 10% buffered formalin. Thick sections (5-6 μm) were made using paraffin embedding method. The sections were stained by hematoxylin and eosin (H&E) and were studied histologically.

Preantral follicles were assigned by the observation of degenerative oocyte and pyknotic granulosa cells. Follicles with`pyknotic granulosa and theca cells, degenerative oocyte and antral cavity filled by numerous apoptotic derbies were characterized as antral atretic follicles. Follicle with intact basal-lamina, zona pellucida and oocyte without cytoplasmic vacuoles, intact germinal vesicle and nucleolus were classified as healthy follicles (40 and 41).

*Statistical analysis*

Data analyses were done using the SPSS 16.0 software package (SPSS Inc., Chicago, IL, USA). The data are reported as mean *± *standard deviation. One way analysis of variance (ANOVA) followed by Tukey test for multiple comparisons were used to assess the variation of the means among the groups. The level of significance for all tests were set at *P* < 0.05. 

## Results

*The Flavonoids and Phenolic contents of A. esculentus*

The results of the present study showed that concentrations of the total phenolics and flavonoids compounds in the *A. esculentus *powder were 141 mg gallic acid/ g and 147 mg quercetine/g in powder respectively.

*Blood glucose level*

A significant increasing of blood glucose level was observed in the HFD diabetic rats compared to the control rats (Figure.1). Administration of *A.esculentus *significantly reduced the blood glucose level in HFD diabetic rats (*P* < 0.05) (Figure.1). The reduction of blood glucose level was not significant between HFD diabetic rats which treated by *A. esculentus*or and Metformin (Figure 1). The *A.esculentus* had no significant effects on blood glucose level in control group rats (Figure 1). 

*Body weight*

At the end of the 5 weeks of HFD diet feeding, the mean weight gain of the HFD diabetic rats did not significantly change (Figure 2), Treatment of HFD diabetic rats by *A.esculentus* or Metformin resulted in weight gain when compared to HFD diabetic rats (*P *< 0.05) (Figure 2). Efficacy of metformin on improvement of weight loss of HFD diabetic rats was similar to *A.esculentus* (Figure 2). No obvious body weight change was observed in control rats treated with *A.esculentus *(Figure.2).

*Serum insulin*

A significant decrease in serum insulin level was observed in the HFD diabetic rats compared to the control rats (*P* < 0.05) (Table 2). Administration of *A.esculentus* caused an significant elevation of serum insulin level in diabetic rats (*P* < 0.05), while Metformin had not obvious effects on serum insulin level of HFD diabetic rats (*P *> 0.05) (Table 2). Serum insulin level had significant effect on insulin level of healthy rats which received *A. esculentus* (*P* < 0.05) (Table 2).

*HOMA-IR*

The HOMA-IR index, which reflects whole body insulin resistance, was increased significantly in HFD diabetic rats compared to control ones (*P* < 0.05) (Table 2). 

Metformin and* A.esculentus*could induce significant reduction in HOMA-IR in HFD diabetic rats compared with untreated HFD diabetic animals (*P *< 0.05) (Table 2). Metformin had more reducing effect on HOMA-IR than *A. esculentus*had (*P* > 0.05) (Table 2). HOMA-IR was decreased in control rats which received* A. esculentus *(Table 2).

*Lipid peroxidation level*

Serum MDA contents was measured to demonstrate the level of lipid peroxidation in all experimental groups. As presented in Table 3, MDA level was increased in the HFD diabetic rats compared to the control rats (*P* < 0.05) (Table 3). MDA levels were decreased in HFD diabetic rats after treatment by* A.esculentus* and Metformin compared to untreated HFD diabetic rats (*P* < 0.05) (Table 3). Administration of *A.esculentus *to control rats resulted in an increase inMDA level compared to the untreaed control rats (*P* < 0.05) (Table 3).

*The antioxidant enzyme activities*

As shown in Table 3, SOD activity has not significantly changed in HFD rats compared to that in normal control rats (Table 3). Administration of* A.esculentus* or metformin in HFD group had not obvious effect on SOD activity (*P* > 0.05) (Table 3). The level of SOD activity did not change in control group that received *A.esculentus*for 30 days (*P* > 0.05) (Table 3). GPX activity has not also significantly changed in the HFD diabetic rats compared to that in the control group (Table 3). Treatment of HFD diabetic rats with *A.esculentus* caused an elevation of GPX level (*P* < 0.05) (Table 3). Serum GPX activity of HFD rats in group IV given metformin was found to be significantly lower compared to that in the HFD rats treated with *A.esculentus* (*P* < 0.05) (Table 3). No significant change was observed in the serum GPX activity of control rats given *A.esculentus* compared with the normal, untreated animals (*P* < 0.05) (Table 3).Significant lower activity of serum catalase was observed in HFD diabetic rats compared to that in control rats (*P* < 0.05) (Table 3). Serum catalase activity was increased in HFD diabetic rats after treatment with *A.esculentus *(*P *< 0.05) (Table 3). The catalase activity in HFD diabetic rats given *A.esculentus *was observed to be significantly higher than that HFD diabetic rat that received metformin(*P* < 0.05) (Table 3). The catalase activity of normal rats treated with *A.esculentus* was observed to be significant compared to that in control animals (*P* < 0.05) (Table 3).

*ovarian expression of apoptotic/antiapoptotic genes*

Expression levels of some genes related to external and internal pathways of apoptosis were analyzed to determine the effect of *A.esculentus *on apoptosis factors dis-regulated by diabetes in ovary. The results showed that the expression of the proapoptotic gene; Bax was significantly higher in ovary of HFD diabetic rats compared to control rats (*P* < 0.05) (Figure 3). In contrast, the mRNA level of antiapoptotic protein; Bcl2 was significantly lower in ovary of HFD diabetic rats compared to the control rats (*P* < 0.05) (Figure 4). Bax transcription was down regulated in HFD diabetic rats after treatment with *A. esculentus* or metformin (*P* < 0.05) (Figure 3). Metformin had more inhibitory effect on ovarian expression of Bax than *A. Esculentus* (*P* < 0.05) (Figure 3). more significant expression of Bcl2 was observed in ovary of HFD diabetic rats treated by *A.Esculentus* or metformin, compared to untreated HFD diabetic rats (*P* < 0.05) (Figure 4). No significant changes were observed in the ovarian expression of Bax and Bcl2 in control rats treated by *A. esculentus *(*P* > 0.05) (Figures 3, 4). The expression of Caspase3 was significantly upregulated in ovary of the HFD diabetic rats (*P* < 0.05) (Figure 5). Treatment of HFD diabetic rats by *A. esculentus *or metformin could inhibit the transcription of Caspase3 in ovary. Metformin had higher reducing effect on ovarian expression of Caspase3 than *A. esculentus *(*P *< 0.05) (Figure 5).

*Ovarian histology*

The histological observation showed numerous healthy follicles at various stages of development and new corpus luteum (Figure 6). Most of these follicles revealed normal features such as intact oocyte and absence of pyknotic granulosa cells and absence of fragmented granulosa cells and cells debris in the antral cavity. (Figure 6). An increased numbers of atretic preantral and antral follicles were observed in the ovary of HFD diabetic rats (Figure 6 and Table 4). Treatment of HFD diabetic rats by* A.esculentus *or Metformin caused a reduction of atretic preantral and antral follicles compared to untreated HFD diabetic rats (Figure 5 and Table 4).* A.esculentus *had more reducing effect on improving atretic follicles compared to metformin. *A.esculentus *had not any side effect on ovaian histology of control rats (Figure 6 and Table 4).

## Discussion

Our results showed that high fat diet feeding and STZ treatment led to an elevation of blood glucose and HOMA-IR levels. These changes were associated with a significant body weight reduction and insulin level. Weight loss in diabetic HFD diabetic rats may be linked to sever hyperglycaemia after inducement of diabetes.

When *A.esculentus* was supplemented in diet of diabetic rats at dose of 200 mg/kg for 30 days, hyperglycaemia and insulin resistance were decreased. These results demonstrated that anti-hyperglycemic activity of *A.esculentus* is comparable to metformin. Treatment of diabetic rats by *A*. *esculentus *and metformin could restore body weight reduction when compared with untreated HFD diabetic rats. In accordance with our findings, Ramachandran *et al.* (42) has reported anti-diabetic activity of okra on alloxan-induced diabetic rats. Sabitha *et al.* (43, 44) has also reported antidiabetic and antihyperlipidemic potential of okra peel and seed powder in STZ-induced diabetic rats. It has been found that administration of peel and seed powder at 100 and 200 mg/kg dose in diabetic rats causes reduction in blood glucose level and increase in body weight. Taken these finding, it is concluded that *A*. *esculentus* treatment can improve body weight reduction and insulin resistance indices of diet induced diabetic animals, supporting that okra may be useful in the treatment of diabetes.

Previous studies reported that consumption of viscous water-soluble dietary fibers reduced postprandial blood glucose by reducing the diffusion of glucose and postponing the absorption and digestion of carbohydrates (45, 46). It has also been reported that different types of dietary, soluble fibers reduce the diffusion of glucose *in-vitro* and also *in-vivo* (45). *A. esculentus* are mucilaginous and a good source of viscous water-soluble fiber and it is assumed that antihyperglycemic effect of okra is due to the presence of large amount of soluble dietary fibers which retard glucose absorption from the gastrointestinal tract (47). To confirm this hypothesis, Khatun *et al.* (45) and Ngoc *et al.* (46) has shown that water soluble fraction of *A*. *esculentus *reduced the absorption of glucose from intestine.

Our results showed that the numbers of antral atretic and preantral follicles in the HFD diabetic rats were higher than control rats that is in agreement with Cordier *et al.* finding (13) which reported that an obese female rabbit model induced by feeding HFD with 83% fat, had a higher number of atretic follicles and a lower number of antral follicles. Honnma *et al.* (48) and Wang *et al.* (4) have also found that follicle growth attenuated in ovaries of fatty rats in concomitant with formation of multiple cysts within the interstitial tissue. They also reported that follicular atresia is accelerated in fatty rats, suggesting that high calorie diet may promote follicle apoptosis. 

Because cell death by apoptosis is the main mechanism involved in follicular atresia (49), the mRNA levels of some apoptosis related genes in ovary of experimental rats were analyzed. Bax, Bcl2, and caspase 3 are the main regulators of apoptosis in follicular cells (50). Bax and caspase3 genes are mainly expressed in atretic follicles while anti-apoptotic BCL2 is abundant in healthy preantral follicles (51). Our results showed the over expression of Bax and caspase3 genes and down regulation of Bcl2 gene in ovary of HFD diabetic rats when compared with control rats. The over expression of caspase3 and Bax in ovary of HFD diabetic rats indicated an involvement of the external apoptosis (death receptor) and internal apoptosis (mitochondrial) pathways in follicular atreisa of animals given high calorie diet (50). 

HFD diabetic rats treated with *A*. *esculentus* and metformin showed fewer preantral and antral atretic follicles and higher new or active follicles than the untreated HFD diabetic rats. Restoration of normal follicular growth was more evident in rats t treated with *A*. *esculentus *than metformin. Daily administration of *A*. *Esculentus *could decrease the ovarian expression of both Bax and caspase3, and increased the mRNA level of ovarian Bcl2. These results confirmed that *A. esculentus* reduced the incidence of follicular apoptosis related to diabetes and that *A*. *esculentus* ingredients may be involved in accelerating follicular maturation by attenuating follicular atresia through an increase in the ratio of antiapoptotic/apoptotic genes in ovary.

It has been found that ovarian follicles can be injured by environmental factors such as hyperglycemia. Granulosa cells are pathologically altered when exposed to hyperglycemia, which has been recently demonstrated in diabetic models (52, 53). Cellular response to high glucose varies and ultimately results in functional changes and cell apoptosis (53). Hyperglycemia causes activation of several proteins involved in apoptotic cell death, including members of the caspase family in various cells (54). Based on this evidence and results from the present study, *A. Esculentus* had a strong glucose reducing capacity and its property may be an important factor for diminishing the apoptotic effects of hyperglycemia on ovary of the *A. esculentus* treated rats.

The interesting finding is increasing of MDA level in diabetic rats which decreased by administration of *A. esculentust *while Metformin had no obvious effect on reducing the serum MDA level in diabetic animals. 

This supportive action of *A. esculentus* in diabetic rats may be induced by its supportive properties against high calorie diet which caused ovarian follicular apoptosis (55). Our results showed no obvious change in the serum activities of SOD and GPX of HFD rats, while CAT activity was reduced in those. Treatment of diabetic rats with *A. esculentus* improved their serum antioxidant status by increasing the activities of GPX and CAT compared to the diabetic group. Metformin had no significant effect on activities of serum GPX, CAT, and SOD in HFD diabetic rats when compared to those treated by *A. esculentus*. 

In agreement with our data, Sabitha *et al.* (2012) found that the administration of different doses of peel and seed of* A. esculentus *increased liver and kidney SOD, CAT, and GPX levels and decreased TBARS levels in diabetic rats (43).

Antioxidants that prevent or limit the damaging effects of oxygen radicals have been reported to have important roles in the female reproductive system and in the pathogenesis of female infertility (56, 21). Based on our findings, antioxidant status of diabetic rats treated with* A.esculentus* powder was improved due to increasing the activities of GPX and CAT and reducing the level of lipid peroxidation. Because ovarian complications related to diabetes display causal relationship with oxidative stress (14), we hypothesized that some beneficial effects of *A. esculentus* against ovarian follicular pathology may be associated with its antioxidant, anti-glucotoxicity, and anti-lipid peroxidation properties. In conclusion our results revealed that *A. esculentus* could improve ovarian structure in HFD diabetic rats. Although molecular mechanisms of the beneficial effects of Okra ingredients on ovarian health needs to be clarified in future, our preliminary study showed that it may, in part, due to attenuation of oxidative stress and inhibition of transcription of apoptosis related genes. 
